# Two Novel Co-Crystals of Naproxen: Comparison of Stability, Solubility and Intermolecular Interaction

**DOI:** 10.3390/ph15070807

**Published:** 2022-06-29

**Authors:** Cheng Xing, Ting Chen, Li Wang, Qi An, Yali Jin, Dezhi Yang, Li Zhang, Guanhua Du, Yang Lu

**Affiliations:** 1 Beijing City Key Laboratory of Polymorphic Drugs, Center of Pharmaceutical Polymorphs, Institute of Materia Medica, Chinese Academy of Medical Sciences and Peking Union Medical College, Beijing 100050, China; xingc@imm.ac.cn (C.X.); chenting1306@163.com (T.C.); wangli_999@126.com (L.W.); a17861121095@163.com (Q.A.); cser_mars@126.com (Y.J.); 2 Beijing City Key Laboratory of Drug Target Identification and Drug Screening, Institute of Materia Medica, Chinese Academy of Medical Sciences and Peking Union Medical College, Beijing 100050, China; dugh@imm.ac.cn

**Keywords:** co-crystal, naproxen, stability, solubility, intermolecular interaction

## Abstract

Two novel co-crystals of naproxen (NPX) were designed and prepared at a stoichiometric ratio of 1:1, namely, naproxen–caprolactam (NPX–CPL) and naproxen–oxymatrine (NPX–OMT). The characteristics of the co-crystals were evaluated in terms of stability and solubility studies. In terms of solubility, in four kinds of solvent systems with different pH, the solubility of NPX–OMT was significantly improved compared with that of NPX, whereas the NPX–CPL showed advantages in acidic solvent systems, indicating that the co-crystals can be applied to concoct preparations depending on therapeutic purposes. Furthermore, the experimental results of the thermal analysis showed that the co-crystal NPX–OMT had better thermal stability than the co-crystal NPX–CPL. Finally, as a complement to the single crystal X-ray diffraction (SC XRD) method, the theoretical calculation based on density functional theory (DFT) was also used to reveal the intermolecular interaction of the co-crystals at the molecular level and visually display the difference between them.

## 1. Introduction

As one of the best-selling drugs in the world, non-steroidal anti-inflammatory drugs (NSAIDs) have been demonstrated to be superior in relieving the pain of patients [1,2]. NSAIDs work by blocking the synthesis enzymes called COX (which are responsible for the production of prostaglandins), namely, COX-1 and COX-2, to suppress pain and inflammation [3,4]. S-Naproxen ((S)-2-(6-Methoxynaphthalen-2-yl) propanoic acid, NPX) belongs to the 2-arylpropionic acid class of NSAIDs and is one of the world’s best-selling drugs with antipyretic and analgesic effects. NPX, belonging to Biopharmaceutical Classification System (BCS) class II, its poor aqueous solubility leads to poor oral bioavailability, which limits its use. NPX is a weak acid with a pKa value of 4.15, causing its disassociation in the gastrointestinal fluids [5,6].

In recent years, the formation of co-crystals has been an effective method to modulate the properties of active pharmaceutical ingredients (APIs) [7,8]. By selecting appropriate co-crystal formers (CCFs) to form non-covalent interactions, such as hydrogen bonding, van der Waals forces, and π-π stacking, co-crystals have the advantage of improving physicochemical properties while keeping their molecular structure unchanged [9,10]. However, intermolecular interactions and crystal packing are utilized at the supramolecular level to create modifications [11]. Thus, pharmaceutical co-crystal technology has been demonstrated to be important in improving the poor solubility of NPX. NPX can form co-crystals or salts with the following CCFs or SFs, namely, nicotinamide [12], isonicotinamide [13] and picolinamide [14], duloxetine [15], and several chiral amino acids, including L-alanine [16], D-alanine, D-tyrosine, and D-tryptophan [17], arginine [18], zwitterionic prolinium [19], tramadol [20], bipyridine and piperazine [21], 4-amino pyridine and 2-amino pyridine [22], urea and thiourea [23]. SC XRD has been used to confirm the structures of most of these co-crystals or salts. Among them, only the solubility of NPX–arginine salt was increased by 25.3 times [18]; other co-crystals have not significantly improved the solubility of naproxen. Compared with the API, the solubility of NPX–nicotinamide co-crystal was just increased by 2.8 times [12], and the intrinsic dissolution rate of NPX–L-alanine increased by two times more than NPX in phosphate buffer pH 7.4 [16]. It was necessary to carry out co-crystal research on NPX, and this prompted us to study co-crystals of NPX with better stability and solubility.

**Figure 1:**
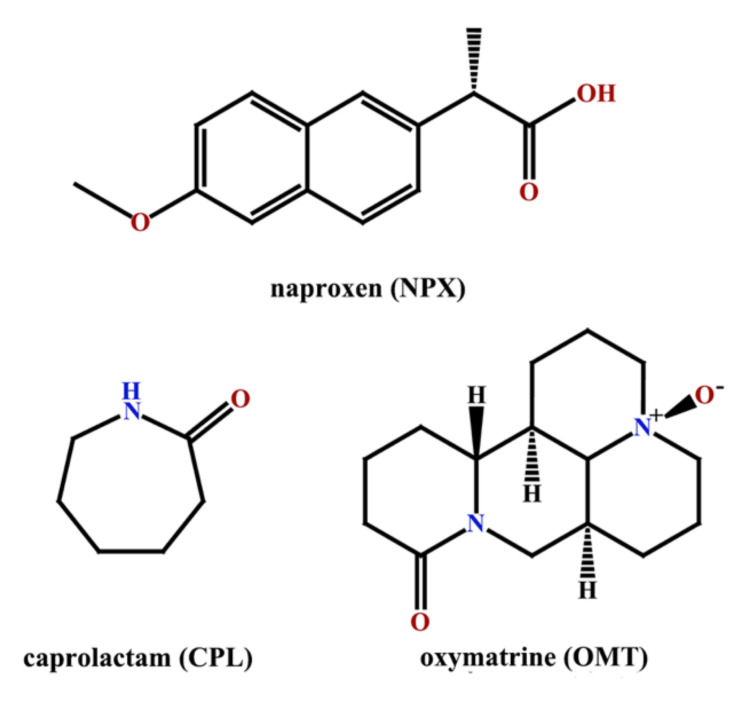
Molecular structures of naproxen, caprolactam and oxymatrine.

The advantages of co-crystals for the improvement of a drug’s ability promote the further design of new co-crystals of NPX. Naproxen is optically active, divided into S-naproxen and R-naproxen. In this work, S-naproxen was used in this study. The co-crystal screening method was used in this study [10]; from a supramolecular perspective, naproxen has an effective functional group (carboxyl group) to form intermolecular hydrogen bonds due to the carboxyl group can act as both hydrogen bond acceptors and hydrogen bond donors. Various CCFs with free carboxylic, hydroxyl or amine groups were selected as potential CCFs [24,25], and finally we successfully obtained two co-crystals of NPX with caprolactam (azepan-2-one, CPL) and oxymatrine ((4R,7aS,13aR,13bR)-10-oxododecahydro-1H,5H-dipyrido [2,1-f:3′,2′,1′-ij] [1,6]naphthyridine 4(41H)-oxide, OMT); moreover, CPL belongs to cyclic imide and OMT belongs to quinoxaline alkaloids. The good news is that OMT has a wide range of pharmacological effects, especially anti-inflammatory [26], which will produce synergistic pharmacological effects on naproxen. The molecular structures of the compounds are shown in Figure 1. These novel co-crystals were characterized by SC XRD, powder X-ray diffraction (PXRD), differential scanning calorimetry (DSC), and Fourier infrared spectroscopy (FT-IR). Furthermore, the stabilities under three conditions were analyzed. Moreover, the solubilities in four solvent systems with different pH, which mimicked the environment in vivo and in pure water mediums, were evaluated. Both of the co-crystals showed excellent stabilities. The solubility of NPX–OMT co-crystal in the four solvent systems was improved, and that of NPX–CPL co-crystal in the acidic solvent system was significantly improved, but the solubility in the neutral solvent system decreased. Finally, a theoretical calculation method based on density functional theory (DFT) was used to analyze the intermolecular interactions of the co-crystals at the molecular level. According to the research results of this paper, the co-crystals NPX–CPL and NPX–OMT can be applied to the subsequent preparation research based on the purpose of the drug design and the site where NPX exerts its therapeutic effect.

## 2. Results and Discussion

The co-crystals NPX–CPL and NPX–OMT in a 1:1 molar ratio were prepared through liquid-assisted griding, and the crystals of them suitable for SC XRD were formulated using the slow evaporation method in different solvents (dichloromethane and acetone, respectively).

### 2.1. SC XRD Analysis

**Figure 2:**
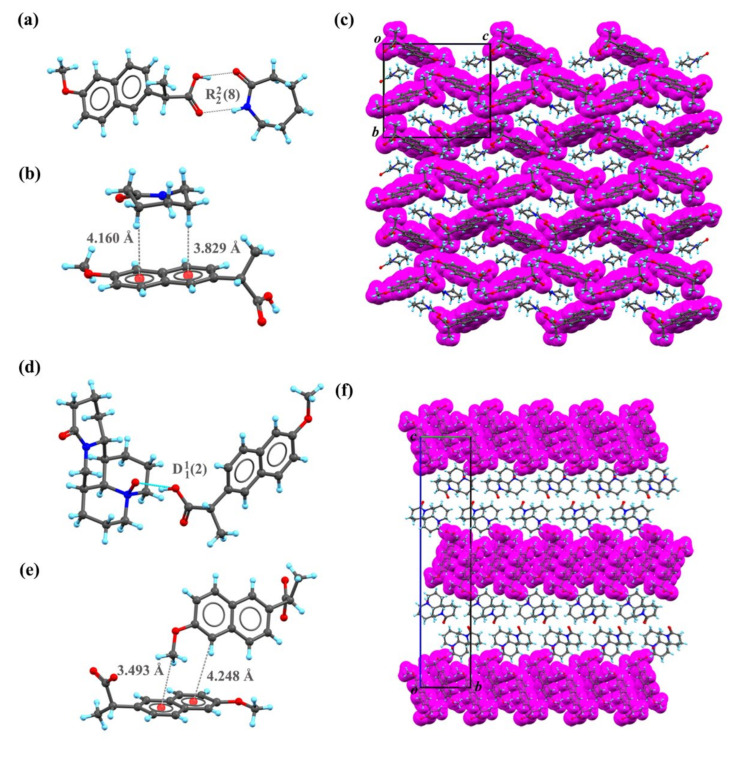
Hydrogen bond schemes and packing pattern of the co-crystals NPX–CPL and NPX–OMT. (**a**) Hydrogen bond scheme of NPX–CPL. (**b**) C-H…π interaction between NPX and CPL. (**c**) Unit-cell packing diagrams of NPX–CPL presented from *a* axis. (**d**) Hydrogen bond scheme of NPX–OMT. (**e**) C-H…π interaction between NPX and OMT. (**f**) Unit-cell packing diagrams of NPX–OMT presented from *a* axis. Note: NPX are drawn with purple space-filled model.

SC XRD was used to pinpoint the exact position of hydrogen bonds and other crystal details [27]. Block-shaped crystals suitable for SC XRD measurement were obtained. The stoichiometric ratio of API to CCFs was 1:1 in NPX–CPL and NPX–OMT. Both were crystallized in the P2_1_2_1_2_1_ space group of the orthorhombic system with Z = 4. Table 1 lists the detailed crystallographic information of the co-crystals. SC XRD analysis was employed to confirm the interactions between API and CCFs and determine whether proton transfer had occurred [28]. Hydrogen bonding interactions were the main drivers of the formation of NPX–CPL and NPX–OMT. The carboxyl hydrogen in NPX and amino hydrogen in CPL were hydrogen bond donors, and the carbonyl oxygen in NPX and CPL acted as hydrogen bond acceptors. In the co-crystal NPX–OMT, the carboxyl hydrogen in NPX was a hydrogen bond donor, and nitroso in OMT acted as a hydrogen bond acceptor. The NPX–OMT co-crystal, determined from SC XRD analysis by evaluating proton location and bond lengths of atoms involved (O(acid)-H: 1.17Å and NO-H: 1.31Å), could be viewed as a “salt-co-crystal continuum”. The proton between the two molecules was “shared” but more on the NPX side [29,30,31]. Table 2 provides the hydrogen bond formation mode and parameters. The hydrogen bonding interactions and packing patterns of the co-crystals are depicted in Figure 2.

In NPX–CPL, the number of molecules in the asymmetric unit was found by SC XRD analysis, one molecule of NPX and one molecule of CPL. Each NPX molecule was linked with one molecule of CPL by a hydrogen bond motif $R_{2}^{2}\left(8\right)$, as shown in Figure 2a. In crystal packing, the CPL molecule interacted with the NPX molecule via C-H⋯π interactions (Figure 2b). These repeated interactions were infinitely arranged along the *a*-axis to form a three-dimensional network (Figure 2c). The number of molecules in the asymmetric unit of NPX–OMT was found by SC XRD analysis, one molecule of NPX and one molecule of OMT. However, the style of packing was very different in the co-crystal NPX–OMT, NPX, and OMT were linked by hydrogen bond motif $D_{1}^{1}\left(2\right)$ (Figure 2d). In crystal packing, two NPX molecules interacted with each other via C-H⋯π interactions to form chains along the a-axis (Figure 2e). Through these interactions, NPX and OMT were connected to form a layered structure (Figure 2f).

### 2.2. PXRD Analysis

**Figure 3:**
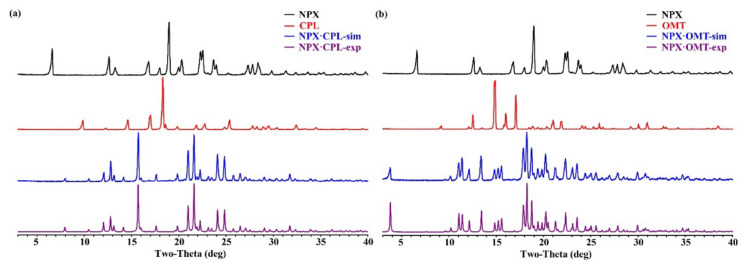
PXRD patterns for API, CCFs, and the corresponding co-crystals. (**a**) NPX–CPL; (**b**) NPX–OMT.

Every crystal structure has its unique PXRD pattern. Both combinations exhibited a different PXRD pattern from the initial pure-components patterns, implying the existence of two new solid forms. In this work, the simulated patterns of both combinations fitted well with the experimental ones. As shown in Figure 3, the API patterns, CCFs, simulated patterns, and experimental patterns of the co-crystals are black, red, blue, and purple, respectively.

### 2.3. DSC Analysis

**Figure 4:**
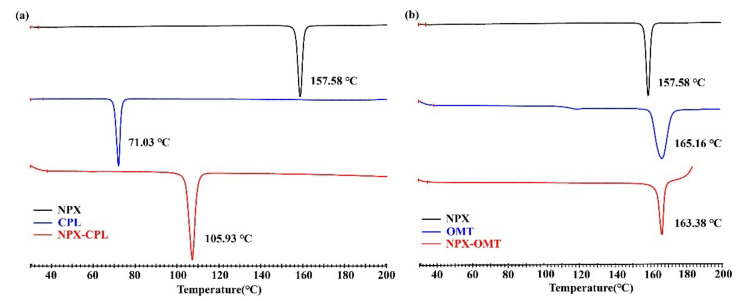
DSC patterns for API, CCFs, and the corresponding co-crystals. (**a**) NPX–CPL; (**b**) NPX–OMT.

Figure 4 displays the thermal characteristics of NPX–CPL, and NPX–OMT obtained through DSC. These endothermic peaks, which corresponded to the melting points of the co-crystals, occurred at significantly different temperatures for NPX, CPL, and OMT, indicating the formation of new phases but not physical mixtures. In general, high melting points indicate thermodynamic stability [32,33]; therefore, the stability of NPX–OMT was higher than that of NPX–CPL. In addition, the endothermic peak values of NPX–CPL, and NPX–OMT were between those of API and CCF. These values were close to that of the corresponding CCF, which indicated that the stability of CCF was extremely relevant to that of the combinations.

### 2.4. IR Analysis

**Figure 5:**
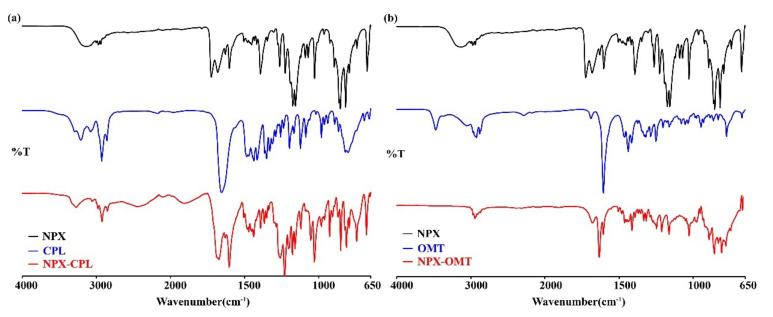
IR characteristics for API, CCFs, and the corresponding co-crystals. (**a**) NPX–CPL; (**b**) NPX–OMT.

FT-IR spectra were recorded for the NPX, CCFs, and co-crystals. SC XRD data showed that both of the co-crystals were formed by the hydrogen bonding, and the IR spectra of the co-crystals were different from API and CCFs, which also confirmed the formation of hydrogen bonding. In fact, the formation of hydrogen bonds can cause the IR wavenumbers to shift toward shorter ones [34]. In NPX–CPL, hydrogen bonds formed not only between the carboxyl hydrogen in NPX and the carbonyl oxygen in CPL but also between the amino hydrogen in CPL and the carbonyl oxygen in NPX. The band attributed to ν_O-H_ at 3143 cm^−1^ in the IR spectrum of NPX black shifted to 3055 cm^−1^, and that attributed to ν_N-H_ at 3292 cm^−1^ in the IR spectrum of CPL blue shifted to 3270 cm^−1^ in the NPX–CPL spectrum. In addition, the ν_C=__O_ stretching band at 1725 cm^−1^ in NPX and 1654 cm^−1^ in the CPL spectra appeared in the NPX–CPL spectrum at 1673 and 1603 cm^−1^, respectively. This finding explained the hydrogen bonding site of NPX with CPL. In NPX–OMT, the ν_O-H_ stretching band at 3143 cm^−1^ in NPX shifts to lower wavenumbers due to the formation of hydrogen bonding. Table 3 shows the main vibrational data of the co-crystals and their preliminary attribution, and Figure 5 displays the IR spectra.

### 2.5. Computation

**Figure 6:**
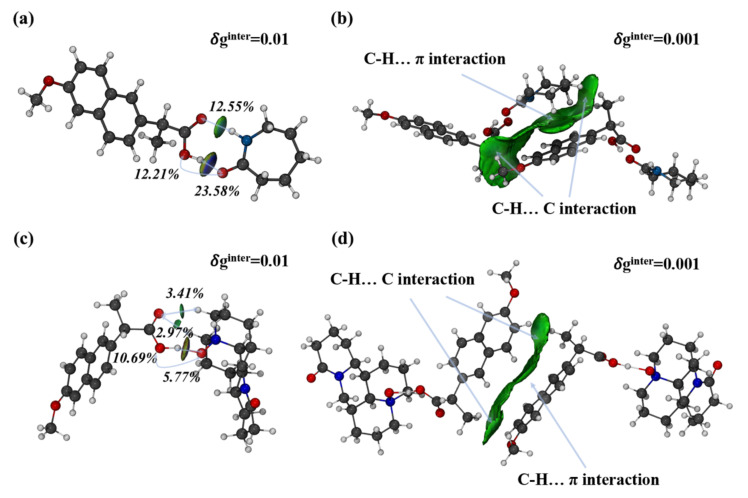
Intermolecular interactions analysis in asymmetric unit (**a**,**c**) and interactions analysis between two asymmetric units (**b**,**d**) of the co-crystals NPX–CPL and NPX–OMT using IGMH method.

Moreover, the IGMH analysis method was utilized to pinpoint classical hydrogen bonds and weak interactions between molecules in the crystal lattice. The molecules in an asymmetric unit in the co-crystals were extracted, and the intermolecular interactions and interactions between two asymmetric units were analyzed by the IGMH method. As shown in Figure 6, the classical hydrogen bonds, such as O-H…O and N-H…O, were elliptic 𝛿ginter isosurfaces of 0.01 a.u. with a blue color in the central area. The darker the shade of blue, the stronger the molecular interaction. Therefore, the hydrogen bond O-H...O was stronger than the hydrogen bond N-H...O. For non-classical hydrogen bonds, such as C-H…O, the interactions were relatively weak. Thus, no blue region was observed on the isosurfaces. In addition, the strength of the hydrogen bond can be determined by the contribution of atomic pairs to intermolecular interactions by the IGMH method. Figure 6a shows that the contribution of atomic pairs O_1_-H_23_ and N_3_-H_21_ was 23.58% and 12.55%, respectively. O-H...O was stronger than the hydrogen bond N-H...O, consistent with the assessment of the isosurface color. Given that the interactions between two asymmetric units were extremely weak in the 𝛿ginter isosurface of 0.01 a.u., the isovalue was adjusted to 0.002 a.u., and the interactions can be revealed by green isosurfaces. The interactions were C-H…π and C-H…C, which were dominated by dispersion effects.

**Figure 7:**
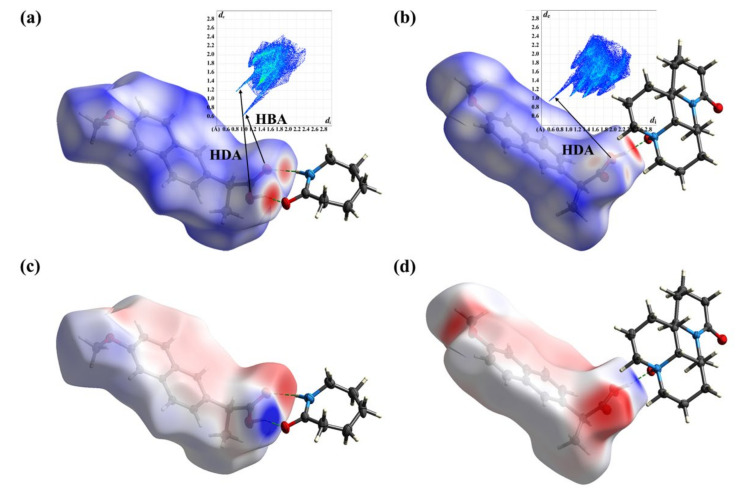
HS, FP and MEPS analysis of NPX–CPL and NPX–OMT. (**a**) HS and FP of NPX–CPL; (**b**) HS and FP of NPX–OMT; (**c**) MEPS of NPX–CPL; (**d**) MEPS of NPX–OMT.

HS, FP, and MEPS analysis are powerful visualization tools for the study of crystal structure. They can clarify the weak interaction between molecules and distinguish the co-crystals. In Figure 7a,b, the deep red spots indicate sites where hydrogen bonds were formed, and the shade of color corresponds to the strength of the interaction. In the NPX–CPL co-crystal, the hydrogen bond O-H...O was evidently stronger than N-H...O. In the NPX–OMT co-crystal, the classical hydrogen bond O-H...O was significantly stronger than the non-classical hydrogen bonds C-H...O and C-H...π. In addition, their FP diagrams revealed that NPX acted as both a hydrogen bond donor (HDA) and acceptor (HBA) in the co-crystal NPX–CPL and in NPX–OMT. NPX only acted as an HDA. In Figure 7c,d, the difference in MEPS between the co-crystal NPX–CPL and NPX–OMT is also significant, i.e., in co-crystal NPX–CPL, the MEPS of NPX appeared as a local positive (red) or negative (blue) region, whereas in NPX–OMT, the MEPS of NPX appeared as a global negative region due to proton transfer and conversion to anion.

Interaction energy can reflect the strength of molecular interactions. The counterpoise-corrected interaction energies of NPX–CPL and NPX–OMT were −11.66 kcal/mole (corrected) and −26.05 kcal/mole (corrected), respectively. Lattice energy can be used to characterize the thermodynamic stability of a crystal structure. The higher the lattice energy, the greater the thermal stability. The calculated Lattice energies of NPX–CPL and NPX–OMT were −24.89 and −33.61 kcal/mol, respectively. The lattice energy of NPX–CPL was significantly lower than that of NPX–OMT, indicating that the thermal stability of NPX–OMT was higher than that of NPX–CPL, which was consistent with the DSC results.

### 2.6. Physical Stability

**Figure 8:**
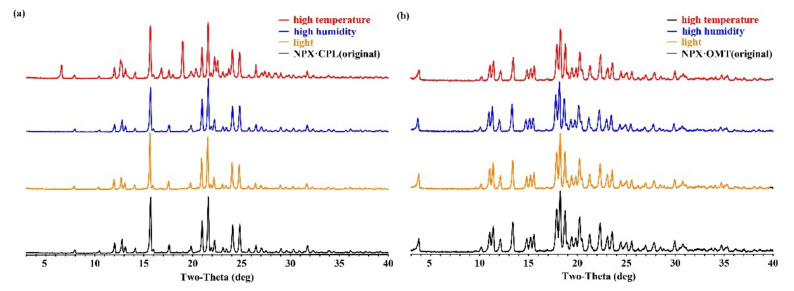
Stability results of co-crystals NPX–CPL (**a**) and NPX–OMT (**b**).

After the formation of co-crystals, the physical and chemical properties changed along with them (Figure 8). For instance, the melting point of NPX–CPL decreased, and that of the NPX–OMT increased. Therefore, stability experiments were carried out to observe whether the co-crystals would undergo phase transition under monitored conditions. The results showed that under the three conditions, the co-crystal NPX–OMT was stable and remained in the same crystal form. The co-crystal NPX–CPL was stable under the high humidity and light conditions and unstable at high temperatures, which decomposed partially into API and CCF. This finding indicates that NPX–OMT can remain stable during transportation and storage, and NPX–CPL needs to be preserved at low temperatures.

### 2.7. Apparent Solubility

**Figure 9:**
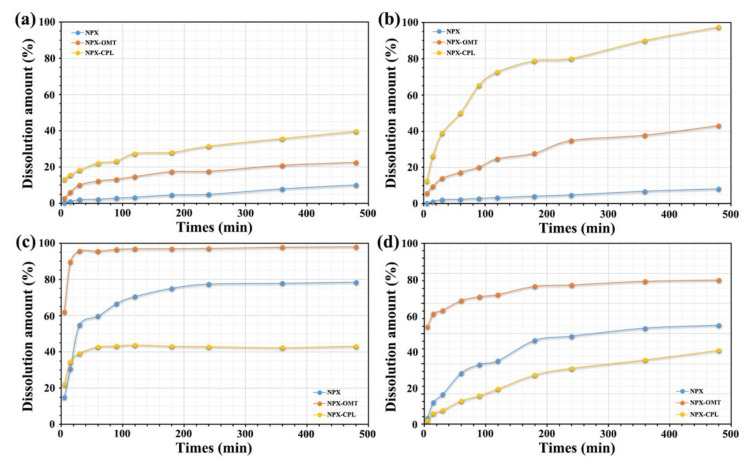
Solubility results of NPX, co-crystals NPX–CPL and NPX–OMT. (**a**) 0.1 mol hydrochloric acid aqueous solution (pH = 1.2); (**b**) acetate buffer (pH = 4.5); (**c**) phosphate buffers (pH = 6.8); (**d**) water (pH = 7.0).

In the acidic solvent system, both of the co-crystals showed the advantages of solubility and dissolution rate (Figure 9), but NPX–CPL showed better results. In 0.1 mol hydrochloric acid aqueous solution (pH = 1.2), the solubility of NPX–CPL increased by 3.9 times, and that of NPX–OMT increased by 2.2 times. In the acetate buffer (pH = 4.5), the solubilities of the co-crystal NPX–CPL and NPX–OMT increased by 11.9 times and 5.3 times, respectively. In the neutral solvent system, NPX–OMT showed a better solubility and dissolution rate than NPX–CPL. In phosphate buffers (pH = 6.8) and water (pH = 7.0), the solubility of NPX–OMT was 1.2 and 1.5 times that of NPX, respectively. However, the solubility of NPX–CPL in water and in phosphate buffer was decreased by 1.8 and 1.3 times, respectively.

The co-crystals exhibited good solubility, and the introduction of CCFs can explain the solubility-enhancing properties of NPX. For molecular crystals, the increase in hydrophilic groups, such as hydroxyl and carboxyl groups, in the structure was conducive to the dissolution of co-crystals. Moreover, the crystal density affects the solubility of the co-crystals to some extent [35]. Therefore, this study achieved its purpose and provided a basis for further research on the reduction of adverse reactions by reducing drug doses [36,37].

## 3. Materials and Methods

### 3.1. Materials

NPX with a purity of >99% and OMT with a purity of >98% were purchased from Wuhan Far Cheng Co-Creation Technology Co., Ltd. (Wuhan, China). CPL with a purity of >99% was obtained commercially from Sinopharm Chemical Reagent Co., Ltd. (Shanghai, China), and all solvents and reagents (analytical grade) were obtained from Beijing Chemical Works (Beijing, China). The molecular structures of the compounds are shown in Figure 1.

### 3.2. Preparation of NPX–CPL Co-Crystal

NPX–CPL was prepared through liquid-assisted griding [38] of the mixture of NPX (23.0 mg) and CPL (11.3 mg) with a 1:1 stoichiometric ratio, which was ground with the addition of 2 mL anhydrous ethanol for about 10 min. Fine block-shaped crystals were obtained by slow evaporation which were suitable for SC XRD. About 80 mg powdered sample of NPX–CPL was dissolved in 6 mL dichloromethane. Subsequently, the solution was filtered, evaporated at 14 °C, and crystallized after 10 days.

### 3.3. Preparation of NPX–OMT Co-Crystal

NPX–OMT was prepared through liquid-assisted griding of the mixture of NPX (23.0 mg) and OMT (26.4 mg) with a 1:1 stoichiometric ratio, which was ground with the addition of 2 mL acetonitrile for about 15 min. Fine block-shaped crystals were obtained by slow evaporation, which were suitable for SC XRD. About 80 mg powdered sample of NPX–OMT was dissolved in 8 mL acetone. Subsequently, the solution was filtered, evaporated at 15 °C, and crystallized after 1 week.

### 3.4. SC XRD

Single crystal X-ray data were measured on a Rigaku MicroMax-002+ CCD diffractometer using Cu Kα radiation (λ = 1.54178 Å) (Rigaku Americas, the Woodlands, TX, USA). All intensity data were collected at 293 K. Data were corrected for absorption effects using the CrystalClear software (Rigaku, USA). Crystal structures were solved by direct methods and refined employing SHELXL and Olex 2 suite of programs, and the final refinements were performed by full-matrix least-squares methods [39,40,41]. All non-hydrogen atoms were refined anisotropically. Hydrogen atoms connected to carbon, nitrogen and oxygen atoms were all placed in idealized positions.

### 3.5. PXRD

PXRD of powder samples was recorded on a Rigaku SmartLab 9 KW diffractometer with Cu Kα radiation (Rigaku, Tokyo, Japan) in the scan range 3°–40° (2θ) at 8°/min scanning rate (step size of 0.02°). Mercury software (Mercury 2022.1.0, Cambridge Crystallo-graphic Data Center, UK) at a starting angle of 3°, a final angle of 40°, was employed to generate simulated PXRD patterns from single crystal structure data [42,43].

### 3.6. DSC

DSC was conducted on DSC 1 (Mettler Toledo, Greifensee, Switzerland), and the data were managed by the STARe Evaluation software 16.30. Approximately 3–8 mg of samples were placed on aluminum pan and heated against blank crimped pan from 30–200 °C at a constant rate of 10 K/min under atmospheric conditions.

### 3.7. IR Spectrum

FT-IR spectra were collected using a Spectrum 400 Fourier transform IR (PerkinElmer, Waltham, MA, USA). Experimental conditions included attenuated total reflection accessory, and the samples were analyzed over the range of 4000–650 cm^−1^ with 16 scanning times having a resolution of 4.000 cm^−1^.

### 3.8. Stability Test

In order to compare the stability of the co-crystals under three conditions: high humidity (90 ± 5%, 25 °C), high temperature (60 °C), and light (4500 ± 500 lx), the stability study was performed in a drug stability test chamber (LHH-SG; Shanghai Blue Leopard Test Equipment, Shanghai, China). The power samples (about 30 mg) were packed in a vial and placed under the three conditions. The samples were analyzed for physical stability after 10 days and compared with the original samples through PXRD.

### 3.9. Apparent Solubility Test

Dissolution studies were carried out on an RC12AD dissolution instrument (Tianda Tianfa, Tianjin, China), following basket method at 100 r·min^−1^ [44]. The dissolution mediums (450 mL) at 37 °C, water (pH 7.0), phosphate buffer (pH 6.8), acetate buffer (pH 4.5), and hydrochloric buffer (pH 1.2), respectively, and solid samples equivalent to 30 mg NPX were added to the dissolution mediums. The sampling points were set at 5, 15, 30, 60, 90, 120, 180, 240, 360, and 480 min. The concentrations of NPX were quantified on an Agilent high-performance liquid chromatography system (Agilent 1200 series, East Brunswick, NJ, USA) with a Welch Materical XB-C18 column (4.6 mm × 250 mm, 6 µm), and the detection wavelength was 235 nm. The mobile phase was prepared with methanol: 0.4% phosphoric acid (70:30); the flow rate was 1 mL·min^−1,^ and column temperature was set at 30 °C.

### 3.10. Theoretical Computation

Theoretical calculations can be used for qualitative and quantitative analysis of the interactions between molecules in co-crystals, and these interactions can be graphically described [45,46]. The theoretical levels of geometry optimizations and single-point energy calculation were B3LYP-D3BJ/6-31G (d, p) and M06-2X/def2-TZVP using Gaussian package [47]. Geometry optimizations were performed only for hydrogen atoms, and the coordinates of heavy atoms were obtained from the experimental of SC XRD [48,49]. The interaction energies of NPX–CPL and NPX–OMT were calculated at m062x -D3/jun-cc-pvtz level using counterpoise corrections method [50]. In addition, the intermolecular interactions existing in the co-crystals NPX–CPL and NPX–OMT were explored by independent gradient model based on Hirshfeld partition (IGMH) method using Multiwfn program [51,52]. The lattice energy calculations were performed for the co-crystals NPX–CPL and NPX–OMT using CRYSTAL17 at the B3LYP level of DFT using the 6-31G (d, p) basis set [53]. Hirshfeld surface (HS), fingerprint (FP), and molecular electrostatic potential surface (MEPS) analysis were carried out by CrystalExplorer (version 21.5) [54].

## 4. Conclusions

In this work, a co-crystal screening method was employed, and finally, two co-crystals, NPX–CPL and NPX–OMT, were prepared using liquid-assisted-griding. Furthermore, fine crystals suitable for SC XRD of both of the co-crystals were obtained by slow evaporation, and their performance was further investigated. Various analytical methods, including SC XRD, PXRD, DSC, and IR, have been carried out to investigate potential differences between the co-crystals and API. The main interaction between API and CCFs was hydrogen bond which was obtained by SC XRD analysis and theoretical calculation methods based on DFT. In addition, the crystal structure analysis showed the presence of NPX–CPL and NPX–OMT chain formation using weak intermolecular C-H⋯π interactions. Physical stability tests demonstrated that NPX–OMT remained stable under three conditions, whereas NPX–CPL was stable under the high humidity and light conditions and unstable at high temperatures. Subsequently, solubility studies were conducted and compared with the previous co-crystal reports of NPX; the solubility of NPX–OMT in four kinds of solvent systems with different pHs all increased compared to that of the API. On the other hand, in the acetate buffer, the solubility of the co-crystal NPX–CPL significantly increased by 11.9 times. Taking account of the results of CCF safety, physical stability tests, and solubility studies, NPX–OMT has a promising prospect and can be selected as an ideal candidate for further study.

## Figures and Tables

**Table 1:** The crystal cell parameters and structure refinement of NPX–CPL, NPX–OMT.

Co-Crystal	NPX–CPL (1:1)	NPX–OMT (1:1)
formula	C_14_H_14_O_3_·C_6_H_11_NO	C_14_H_14_O_3_ ·C_15_H_24_N_2_O_2_
crystal size (mm)	0.15 × 0.280 × 0.340	0.15 × 0.170 × 0.300
molecular weight	343.17	494.27
Temperature (K)	293(2)	293(2)
crystal system	orthorhombic	orthorhombic
space group	P2_1_2_1_2_1_	P2_1_2_1_2_1_
α (Å)	7.565(1)	5.867(1)
b (Å)	14.642(1)	9.343(1)
c (Å)	16.833(1)	46.507(2)
a (deg)	90	90
β (deg)	90	90
γ (deg)	90	90
volume (Å3)	1864.69(6)	2549.39(16)
Z	4	4
density (g/cm³)	1.223	1.289
R1 (*I* > 2σ(*I*))	0.056	0.079
wR2 (*I* > 3σ(I))	0.154	0.212
goodness-of-fit on F^2^	1.050	1.084
Completeness (%)	99.9	99.5
CCDC deposition no.	2,172,223	2,172,224

**Table 2:** Parameters (Å, Degree) of Main Hydrogen Bonds for NPX–CPL, NPX–OMT.

Co-Crystal	D–H…A	D…A	_∠_DHA
NPX–CPL	^a^ O_3__NPX–_H_3NPX_…O_1CPL_	1.75	155.35
	^b^ N_2CPL_-H_2CPL_…O_2NPX_	2.165	167.11
NPX–OMT	O_2CPL_ -H_2CPL_…O_2OMT_	1.697	157.72
	O_2CPL_ -H_2CPL_…N_2OMT_	2.634	165.05

Symmetry Code: ^a^ −x + 3/2, −y + 1, z + 1/2; ^b^ −x + 3/2, −y + 1, z − 1/2.

**Table 3:** Main Vibrational Frequency (cm^−1^) with Tentative Assignments for NPX–CPL, NPX–OMT.

Vibrational Data		Vibrational Assignment
NPX–CPL	NPX–OMT	
3270	–	–NH stretch
3055	–	–COOH stretch
2978	2968	–CH_3_ stretch
1451	1439	–CH_3_ stretch
2924	2945	–CH_2_– stretch
2850	–	–CH_2_– stretch
1673	1679	C=O stretch
1630	–	–NH bend
1439	–	–COOH bend
1392	1392	–CH_3_ bend
1472	1462	–CH_2_ bend
744	667	Ar-H bend

## Data Availability

CCDC 2,172,223 and 2,172,224 contain the supplementary crystallographic data for this paper. These data can be obtained free of charge via www.ccdc.cam.ac.uk/data_request/cif, or by emailing data_request@ccdc.cam.ac.uk, or by contacting The Cambridge Crystallographic Data Centre, 12 Union Road, Cambridge CB2 1EZ, UK; fax: +441223 336033.
